# Sinus Augmentation with Biomimetic Nanostructured Matrix: Tomographic, Radiological, Histological and Histomorphometrical Results after 6 Months in Humans

**DOI:** 10.3389/fphys.2017.00565

**Published:** 2017-08-03

**Authors:** Antonio Scarano, Felice Lorusso, Giorgio Staiti, Bruna Sinjari, Anna Tampieri, Carmen Mortellaro

**Affiliations:** ^1^Department of Medical, Oral and Biotechnological Sciences and CeSi-MeT, University of Chieti-Pescara Chieti, Italy; ^2^Private Practice Torino, Italy; ^3^Bioceramics and Bio-hybrid Composites Senior Affiliated Member Methodist Hospital Research Institute Houston, TX, United States; ^4^Department of Health Sciences Oral Surgery Unit, University of Eastern Piedmont Novara, Italy

**Keywords:** biomimetic, Mg-MgHA/collagen, sinus augmentation, biomaterials, bone healing

## Abstract

**Background:** Many bone substitutes have been applied for sinus regeneration procedures, such as autogenous bone, inorganic bovine bone, porous and resorbable hydroxyapatite, tricalcium phosphate, bioactive glass, and blood clots. The aim of the present study was a tomographic, histological and histomorphometrical evaluation in humans, of specimens retrieved from sinuses augmented with MgHA/collagen-based scaffolds, after a healing period of 6 months.

**Materials and Methods:** Eleven healthy patients and a total of 15 sinuses were included in this study. The maxillary sinuses were filled with commercial MgHA/collagen-based scaffolds (RegenOss) with a porous three-dimensional (3D) structure (Fin-Ceramica Faenza S.p.A., Faenza, Italy). These grafts have a composite design, that replicate the organization of bone structure, obtained by a technique in which a specific hybrid organic–inorganic composite is spontaneously built by a biological mechanism. The CBCT scans were done before the procedure, after the surgical protocol (T1), and 6 months after sinus surgery (T2) for implantology. Bone specimens were stored in 10% formalin solution, embedded in a glycolmethacrylate resin and sectioned by a high-precision diamond disc. Histologic and histomorphometric analysis were carried out to evaluate the graft reabsorption and bone healing.

**Results:** The mean volume after graft elevation, calculated for each of the 15 sinuses, was 2,906 mm^3^ in the immediate postoperative period (5–7 days), ranging from 2,148.8 to 3,146.4 mm^3^. In the late postoperative period (6 months) it was 2,806.7 mm^3^, ranging from 2,010.9 to 3,008.9 mm^3^. The sinuses were completely healed and no residual MgHA/collagen-based scaffolds were visible. Osteoblasts appeared actively secreting bone matrix and marrow spaces contained moderate numbers of stromal cells and vascular network. Osteoblasts were observed actively secreting osteoid matrix. The tissues present in the samples were composed of 1.9 ± 1.9% of lamellar bone, 36 ± 1% of woven bone and 58 ± 3.8% of marrow spaces.

**Conclusion:** Mg-MgHA/collagen-based scaffolds can successfully be used for sinus augmentation procedures.

## Introduction

Insufficient bone height, following tooth extractions and the pneumatization of the maxillary sinus in the lateral part of the maxilla, is an impediment to dental implant primary stability and a contraindication for implant surgery. In fact, in this situation oral rehabilitation with dental implants is often difficult and there is a high risk of implant displacement/migration into the maxillary, as has previously been reported. The augmentation of bone volume in posterior maxilla atrophy can be performed using a sinus lifting technique described by Tatum in 1986 (Tatum, 1986), allowing for about 6–8 months healing before implant insertion (Del Fabbro et al., 2004). Different biomaterials are currently used in bone regeneration and can be classified into four groups according to their origin: autogenic (bone originating from the same patient), allogenic (bone originating from another person), xenogenic (bone originating from another species) and synthetic (with no biological origin) (Scarano et al., 2006). The features of a bone substitute are critical factors for the success of bone augmentation.

Autogenous bone is considered ideal (Hallman et al., 2002; Samartzis et al., 2005). Donor sites for these techniques are usually the iliac crest for bilateral procedure and the oral cavity for unilateral sinus regeneration. Patients may evaluate the second surgical intervention in the donor area uncomfortable and may prefer the use of bone substitutes for the procedure. Biomaterial is a key component for the success of implants inserted into the grafted maxillary sinus. There is concern that some biomaterials may cause a foreign body reaction and the ideal material for sinus augmentation is still under debate (Scarano et al., 2017a).

Nevertheless, the application of xenografts has been associated with the permanency of residual material, due to their slow rate of reabsorption (Scarano et al., 2006) and the waiting time necessary for the bone to heal after biomaterial application, limiting their use in surgery. These results may be caused by the fact that structural and composition properties of these materials do not resemble those of natural bone. For this reason, many researchers have studied new synthetic grafting materials that promote bone formation with faster resorption processes and new combinations of osteoinductive scaffolds and stem cell-based protocol. Tissue engineering in the field of bone regeneration requires:

- biocompatible scaffold adhesion, diapedesis, proliferation, and differentiation of stem cells;
- an appropriate stem cell source for the deposition of new bone.

In an attempt to increase graft resorption and reduce healing time before implantation, while avoiding autologous bone harvesting, biomimetic Mg-MgHA/collagen-based scaffolds with a sinus augmentation procedure were used in this study. Hydroxyapatite (HA) is an osteoconductive, synthetic bioactive material, without osteoinductive properties, which limits its clinical use. The collagen has been added because it is present in the extracellular matrix of bone, performing the function of support by giving structural support to resident cells, osteoconductivity, biocompatibility, and ductility to the bone. Since Collagen type I is one of the proteins that play critical roles in bone mineralization, it can be the prime candidate material for realizing tissue-engineered grafts. The association of Hydroxyapatite and collagen type I (HA/Collagen) were used to improve osteoblast differentiation (Ramírez-Rodríguez et al., 2016). Magnesium (Mg) was added to HA for its positive role in bone healing. Its role is critical in the metabolism and turnover of bone (Toba et al., 2000).

These grafts have a composite architecture, mimicking the complex hierarchically organized bone structure, obtained through a proprietary technique in which a specific hybrid organic–inorganic composite is spontaneously built, driven by a biological mechanism. We hypothesize that this biomaterial is completely resorbable. In fact, many biomaterials act only as passive scaffolding, so insufficient bone remodeling occurs when bone regeneration mixes with grafts that do not resemble those of natural bone. Various studies have shown the osteoregenerative properties of Mg-MgHA/collagen in bone regeneration, but data on its use in maxillary sinus lifts in humans are not available yet (Berardinelli et al., 2013; Ramírez-Rodríguez et al., 2016). The aim was a radiologic, histological and histomorphometrical evaluation of a biomimetic nanostructure applied for sinus augmentation after a healing period of 6 months.

## Materials and methods

### Biomaterial

For the present research, a commercial MgHA/collagen-based scaffold, the RegenOss® (Fin-Ceramica Faenza S.p.A., Faenza, Italy), was used. The device is a commercially available, porous, three-dimensional composite bone substitute made of type I collagen fibers, in which nano-sized (10–20 nm) crystals of biomimetic Mg-doped hydroxyapatite (Mg-HA) are nucleated in a 40/60 wt ratio.

The composite material was produced following a biomineralization approach allowing for the formation of a bio inspired nanostructure consisting of nano-apatite crystals (Mg-HA) uniformly distributed in the bio-polymeric collagen matrix. The device is manufactured being capable of reproducing the composition and the anatomical structure of the bone tissue, as it occurs in the biological process of neo-ossification. The organic component, working as a matrix mediating the mineralization process, was type I collagen extracted from equine Achille's tendon and was supplied by OPOCRIN Spa (Corlo di Formigine (MO), Italy) in a 1wt % suspension in an acetate buffer solution, pH 3.5. The apatitic phase (Mg-HA) was synthetized directly on the collagen molecules by a neutralization process where an acid solution containing suspended type I collagen in phosphoric acid (H_3_PO_4_, purity ≥ 85 wt%; Sigma Aldrich) was added drop-wise to a basic solution containing calcium hydroxide (Ca(OH)_2_, purity ≥ 95 wt%; Sigma Aldrich) and magnesium chloride hexahydrate (MgCl_2_▾6H_2_O, purity ≥ 99 wt%; Sigma Aldrich). This method enables the manufacture of hybrid composites Mg-HA/Coll with a composition of 40/60 wt % obtained in form of a gel that was freeze-dried to achieve a porous device.

The device has a high safety profile highlighted by toxicological studies carried out in accordance with the laws and regulations in force concerning Class III medical devices. Once the tissue regeneration process has been completed, the device is able to undergo resorption. The mineralized structure is manufactured by nucleating bonelike nanostructured nonstoichiometric hydroxyapatite into self-assembling collagen fibers, as it occurs in the biological process of neo-ossification (Palarie et al., 2012).

These grafts have a composite architecture, mimicking the complex hierarchically organized bone structure, obtained through a proprietary technique in which a specific hybrid organic–inorganic composite is spontaneously built, driven by a biological mechanism. In particular, the biomaterial consists of a combination of type I collagen (30%) and MgHA (70%). It is synthesized using a standardized procedure that begins from an atelocollagen aqueous solution in acetic acid (1%, w/w), obtained from equine tendon (Opocrin S.p.A., Modena, Italy).

### Surgical procedure

**Figure 1:**
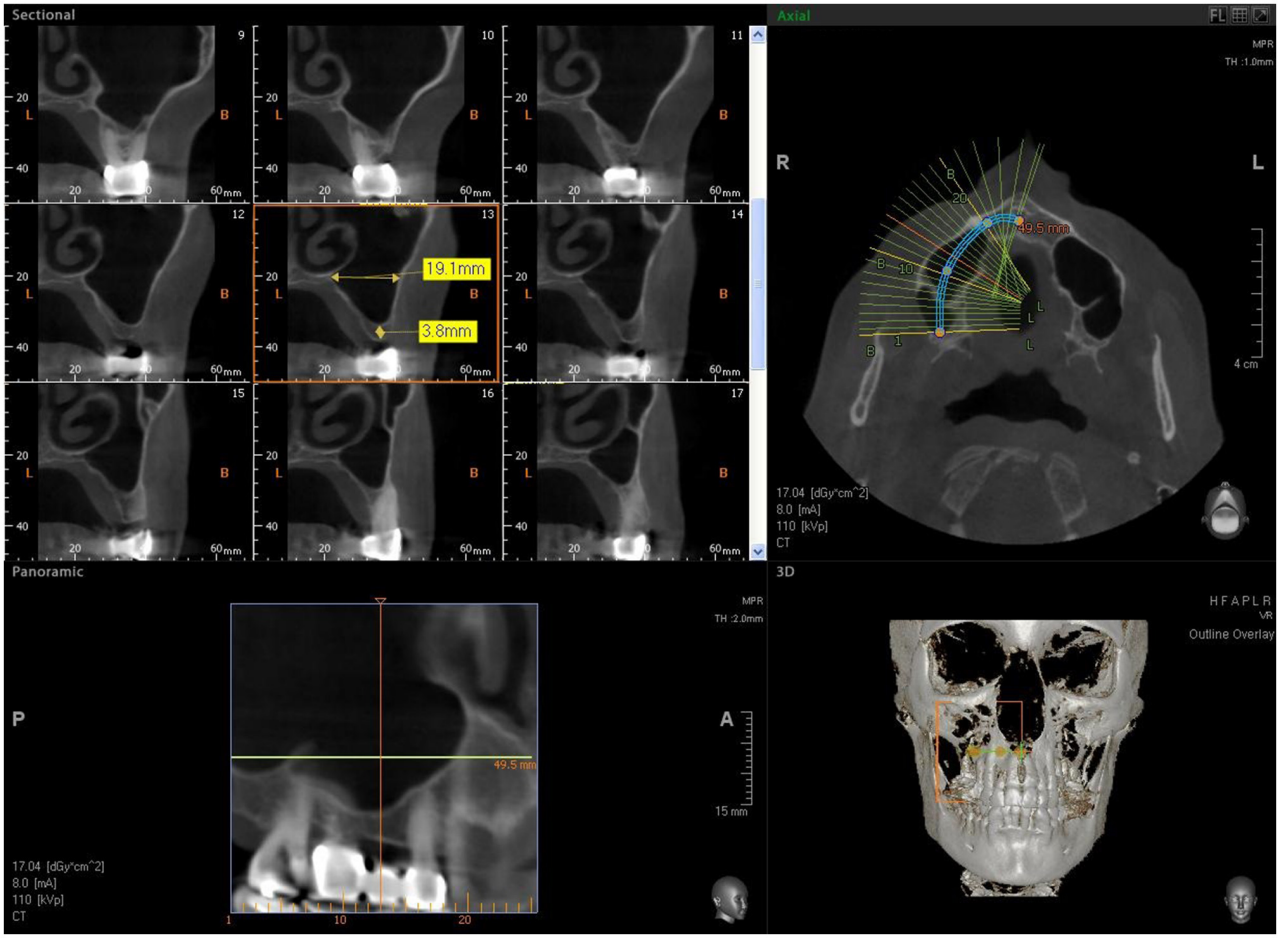
CBCT image showing reduced bone height in the sinus area with a residual alveolar ridge height between 2 and 3 mm.

This study was performed following the principles of the Declaration of Helsinki regarding research on humans; all patients gave a written informed consent to the treatment and study recruiting. The study was approved by the Inter Institutional Ethics Committee of University of Chieti-Pescara, Chieti, Italy. Eleven healthy patients (mean age: 52 years; range 48–65 years) without significant medical anamnesis, 7 women and 4 men, all non-smokers, were recruited as candidates for sinus augmentation and implant rehabilitation. They were treated in the Outpatient Department of Medical, Oral and Biotechnological Sciences of the University of Chieti-Pescara, Chieti, Italy. All sinus lifts were performed by a single surgeon. The inclusion criteria were:

- totally or partially edentulous
- unilateral or bilateral tooth loss (premolar or molar)
- severe atrophy (bone height: between 2 and 3 mm) (Figure 1).
  The exclusion criteria were:
- severe disease, uncontrolled diabetes and smoking,
- head and neck radio or chemotherapy,
- uncontrolled periodontal disease, sinus pathology, or the presence of any dental roots in the sinus area.

At the initial visit, all subjects were clinically examined and radiographs were carried out for diagnostic evaluation; then the patients were scheduled for surgery procedures including sinus augmentation and implant insertion. They were informed about the surgical protocols and full cooperation was requested during the procedures. Before surgery the patients were subjected to applications of chlorhexidine digluconate solution 0.2% for 2 min to obtain lower bacterial load and local anesthesia was given with Articaine® (Ubistesin 4%—Espe Dental AG Seefeld, Germany) associated with epinephrine (1:100.000). A modified triangular flap, without anterior release, recently described by Scarano et al. was used. The incision was made horizontally on the top of the alveolar ridge extending mesially if the patient was edentulous, in the presence of teeth the incision was continued by a sulcular incision starting near the mesiobuccal edge of the teeth extending up to the midpoint of the buccal sulcus of the canine, without cutting the interdental papilla. Full thickness flaps were detached to expose the bone ridge and the lateral wall of the sinus. A trap door was made in the lateral sinus wall with a piezoelectric device (Piezosurgery, Mectron, Carasco, Italy) under cold (4–5°C) sterile saline irrigation solution and the bone door was rotated inward and upward with a top hinge to a horizontal position. The detachment and elevation of the sinus membrane was accomplished by initially exposing and mobilizing by ultrasonic device, followed by hand instrumentation to further elevate it along the medial bone wall.

**Figure 2:**
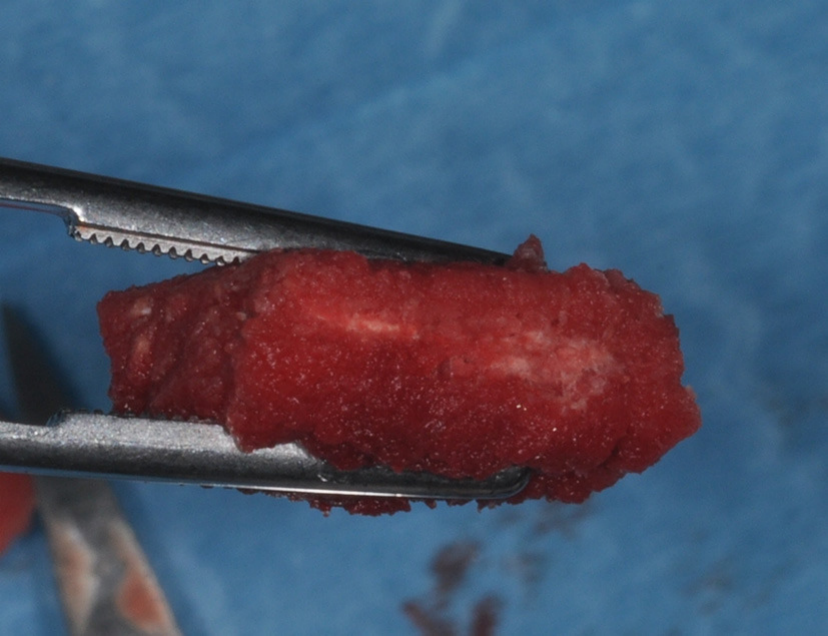
During graft placement the blood molecules and cells promoting bone formation are quickly absorbed.

**Figure 3:**
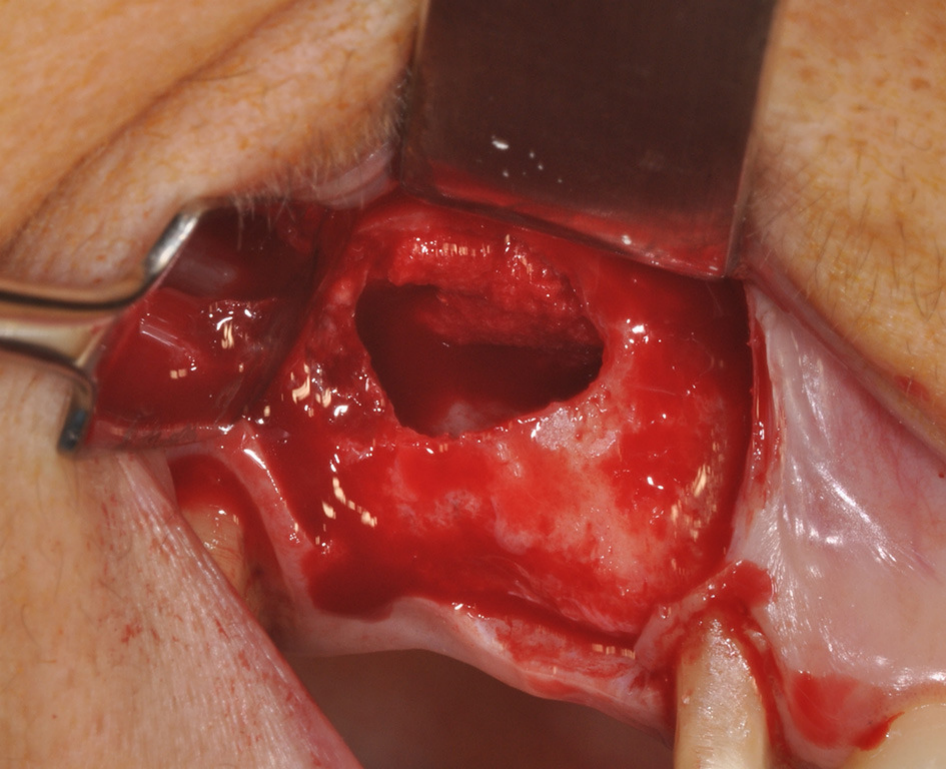
The maxillary sinus lateral wall is exposed and a bone window is cut out.

**Figure 4:**
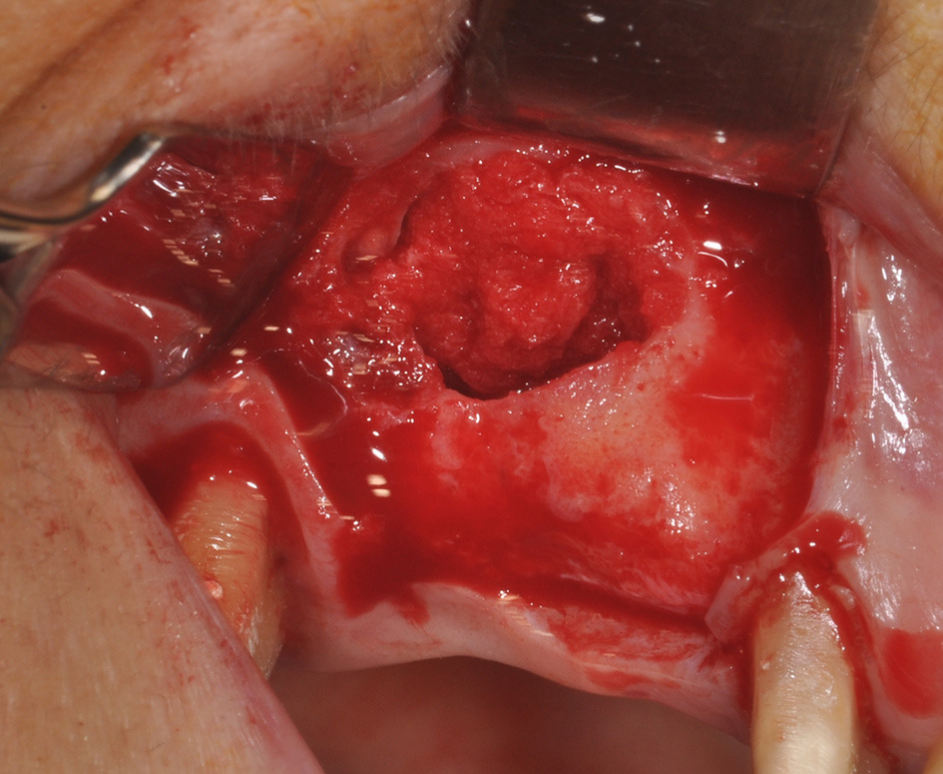
The maxillary sinus filled with commercial MgHA/collagen-based scaffold.

**Figure 5:**
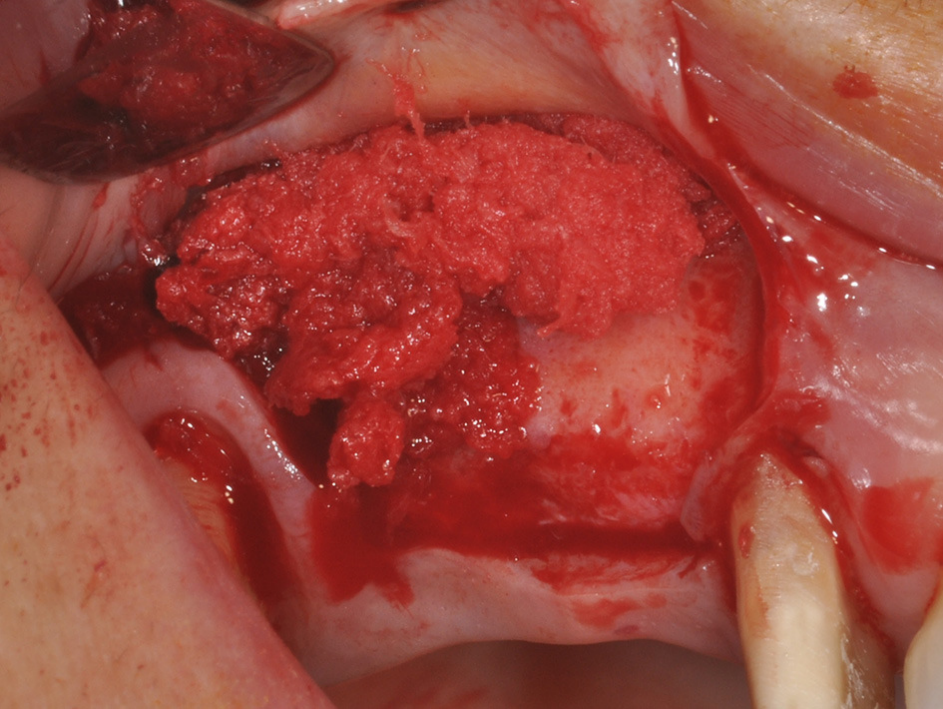
During sinus filling, the scaffold can be easily adapted to the dimension and shape of the sinus, saving time, and improving sinus filling.

**Figure 6:**
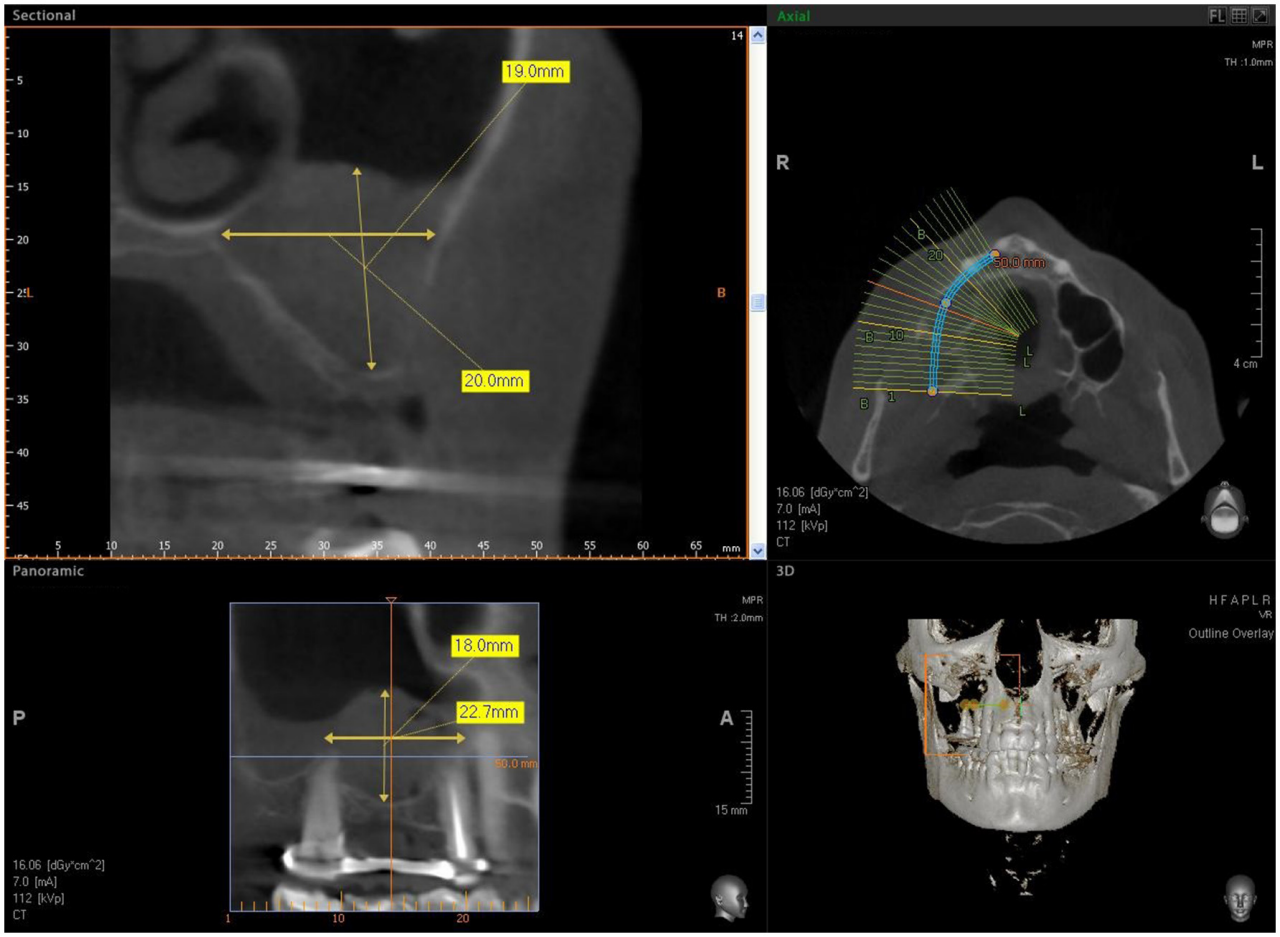
CBCT after sinus augmentation, the grafted biomaterial is clearly distinguishable from the remaining original bone in regard to its density and structure. The biomaterial is well circumscribed with no scattered particles in the sinus.

Seven patients were treated for unilateral sinus augmentation, while in 4 patients the procedure was bilateral for a total 15 maxillary sinuses. The maxillary sinuses were filled with commercial MgHA/collagen-based scaffold (RegenOss®) (Figures 2–5) with a porous three-dimensional structure (3D) (Fin-Ceramica Faenza S.p.A., Faenza, Italy). Thirty-three implants (Bone System, Milano, Italy) were positioned in the treated sinuses after a healing phase of 6 months. Cone Beam Computed Tomography evaluation (CBCT) (VatechIpax 3D PCH-6500, Fort Lee, NJ USA) was performed for preoperative and post-surgical sinus augmentation. DICOM data were elaborated with Ez3D Plus Software (EZ3D Plus, VATECH Global Fort Lee, NJ USA) to elaborate 3D model specimens and find the perfect position and alignment of sinus and biomaterial scaffolds with the bone itself. The CBCT scans were conducted before surgery (Figure 1), to diagnose the bone, immediately after surgery (T1), and 6 months after sinus grafting (T2) (Figure 6) since this period was recommended by the manufacturer for implant insertion. The CBCT scans were obtained with 1.0 mm in thickness and 0.2 mm interval under 110 kVp and 8 mA with a very low dosage. After selection of the appropriate area, using a specific tool and 3D reconstruction by an experienced radiologist, the software measured the volume.

### Undecalcified specimen preparation and histomorphometry

**Figure 7:**
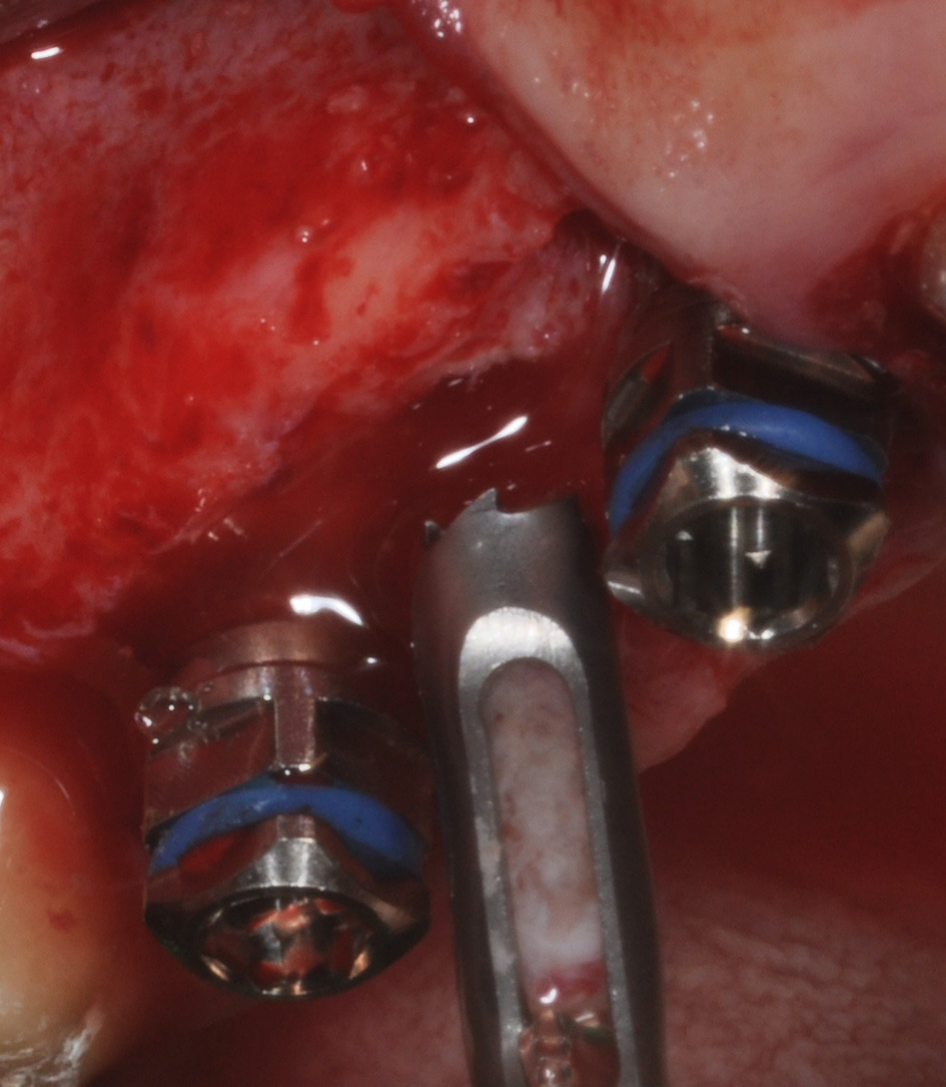
After 6 months of healing: Maxillary sinus augmentation performed with biomaterial. During implant placement, the maxillary sinus lateral wall is completely filled by new bone and bone core retrieval with a trephine bur.

Bone specimens were obtained by a trephine bur with a 2 mm internal diameter and 13 mm length, stored in 10% formalin solution and treated to obtain thin sections (Figure 7); the next phase consisted in processing the samples by Precise 1 Automated System (Assing, Rome, Italy). The samples were dehydrated in a graduated series of ethanol, embedded in a glycolmethacrylate resin (Technovit 7200 VLC, Kulzer, Wehrheim, Germany) and sectioned longitudinally at about 150 μm by a high-precision diamond disc. The sections were also thinned to 30 μm by a grinding machine (Precise 1 Automated System, Assing, Rome, Italy). Three slides were collected for each specimen and stained with toluidine blue for transmitted light microscopy examination (Leitz Laborlux, Leitz, Wetzlar, Germany).

Histomorphometry was used to evaluate the residual graft material, the percentages of newly-formed bone and marrow spaces. This evaluation was carried out by observation through a microscope (Laborlux S, Leitz, Wetzlar, Germany), connected to a high definition camera (3CCD, JVC KY-F55B, JVC®, Yokohama, Japan) and Personal Computer interface (Intel Pentium III 1200 MMX, Intel®, Santa Clara, CA, USA). This equipment was linked to a digitizing pad (Matrix Vision GmbH, Oppenweiler, Germany) and a histometry software with image capturing applications (Image-Pro Plus 4.5, Media Cybernetics Inc., Immagini & Computer Snc Milano, Italy). Two separate histologists evaluated the results and found few differences.

## Results

The biomaterial shapes perfectly to fit the anatomic curvature of the graft sinus. During surgery, this osteostimulative and biodegradable scaffold can be easily adapted to the dimension and shape of the sinus, saving time and improving sinus filling. During graft placement, it can quickly adsorb the blood molecules and cells, promoting bone formation. Its architecture favors cell attachment and proliferation. The grafted biomaterial was clearly distinguishable from the remaining original bone, due to its density and structure (Figures 3–5). The mean volume after graft elevation, calculated for each of the 15 sinuses, was 2,906 mm^3^in the immediate postoperative period (5–7 days), ranging from 2,148.8 to 3,146.4 mm^3^. In the late postoperative period (6 months) it was 2,806.7 mm^3^, ranging from 2,010.9 to 3,008.9 mm^3^ (Figure 6). A total of 45 CT scans of the sinus augmentation of the 15 sinuses were evaluated (Table 1). No perforation of the sinus membrane was evident in 12 sinuses, while in 3 sinuses a small perforation was evident. No acute infection, nor pain nor fever were observed.

**Table 1:** Volume graft at T0 and at 6 months from the surgery.

**N° sinus**	**Volume after graft elevation (mm^3^)**	**Volume after 6 months (mm^3^)**
#1	2,800	2,810
#2	3,120	2,806
#3	2,148	2,011
#4	2,253	2,220
#5	3,001	2,890
#6	3,147	2,960
#7	2,974	2,930
#8	3,101	3,040
#9	3,010	2,820
#10	2,980	2,890
#11	2,909	2,890
#12	3,103	2,992
#13	3,120	2,813
#14	3,010	2,901
#15	2,929	2,830
Mean volume	2,907 mm^3^	2,806 mm^3^
Range	2,148.8 to 3,146.4 mm^3^	2,010.9–3,008.9 mm^3^

**Figure 8:**
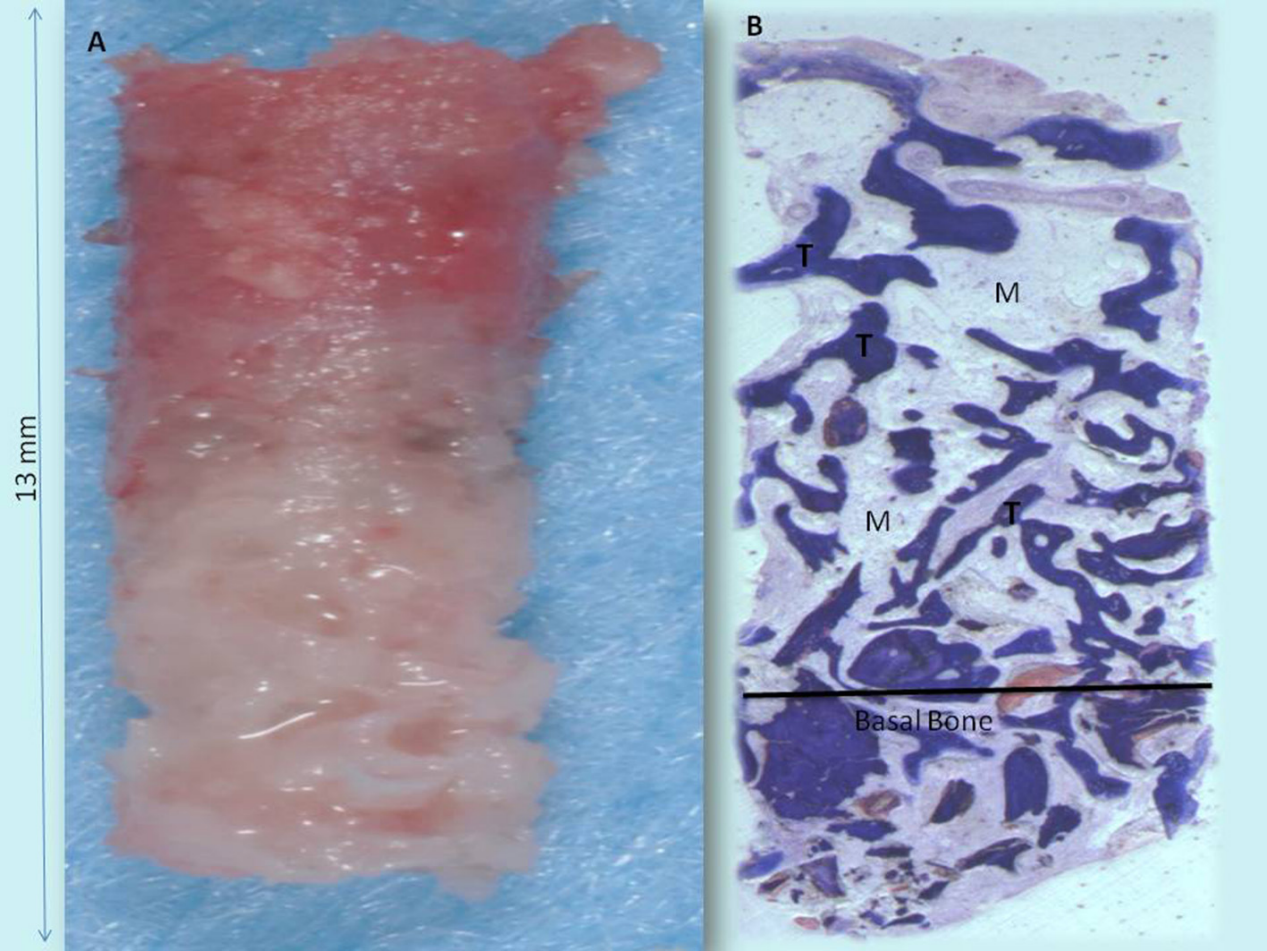
**(A)** Bone core biopsy carried out with a small trephine. **(B)** The sinus is filled by the newly formed trabecular bone (T) with wide marrow spaces (M), while lamellar bone and haversian system were not present. No residual biomaterials were present. Toluidine blue 3X.

**Figure 9:**
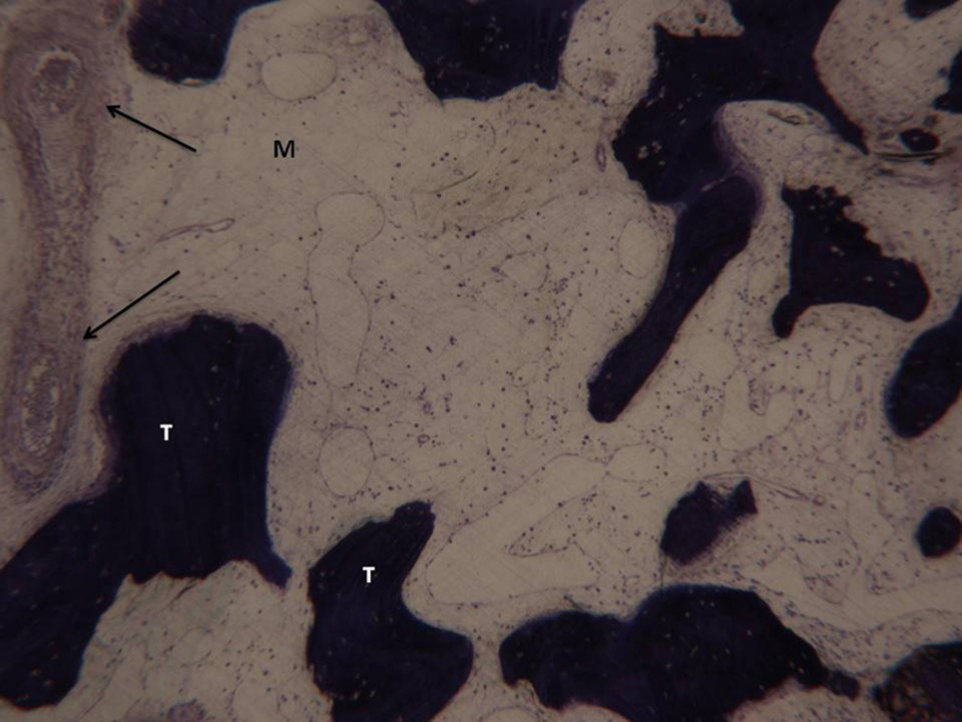
In the marrow space no pathological inflammatory cell infiltrate is present. Neither foreign body reaction cells nor multinucleated giant cells were observed. A small trabecular bone (T) with a large marrow space (M) and vessels is present (arrows). Toluidine blue 100X.

**Figure 10:**
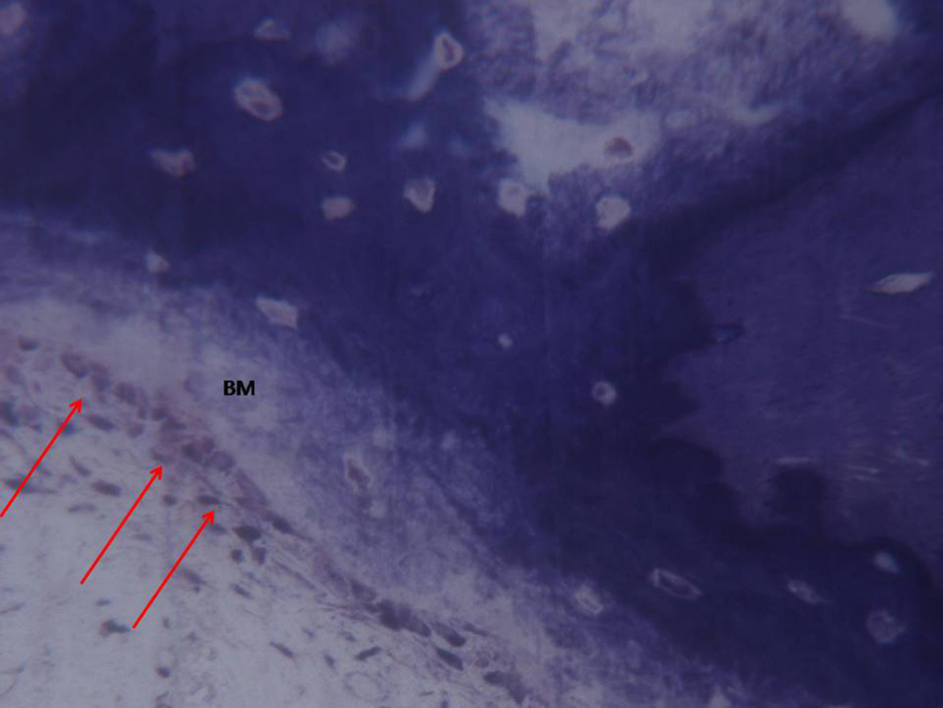
A higher magnification of the previous image. Osteoblasts (arrows) appeared actively secreting bone matrix (BM). Toluidine blue 200X.

In all cases, bone augmentation showed hyper density in the immediate postoperative period and late postoperative period, with more density than native bone at both times. The statistical analysis demonstrated a significant difference of volume change (*P* = 0.001229). At low magnification, trabecular mature bone was observed. Osteoid material was found only around some of the particles. In all specimens, no pathological inflammatory cell infiltrate was present. No foreign body reactions were present. The biomaterials were completely resorbed. No epithelial cells or connective tissue were found in the retrieved specimens. Prominent woven and mature bone was observed. Mature bone deriving from the endosteal surface filled the external portion of the bone sinus. The periphery and central portion of the cavities showed mineralized new bone formation. The sinuses were completely healed and no particles or MgHA/collagen-based scaffolds were visible (Figures 8, 9). Osteoid matrix actively secreted by osteoblasts (Figure 10) andmoderate numbers of marrow stromal cells and vascular network contained in marrow spaceswere observed. In particular, seams of osteoblasts and unmineralized matrix with collagen fibrils in areas of new bone apposition were observed. The tissues present in the sample were composed of 1.9 ± 1.9% of lamellar bone, 36 ± 1% of woven bone and 58 ± 3.8% of marrow spaces (Table 2).

**Table 2:** Percentage of lamellar bone, woven bone and marrow space.

**N° sinus**	**Lamellar bone**	**Woven bone**	**Marrow spaces**
1	4	35	61
2	3	35	54
3	3	34	61
4	2	35	59
5	0	37	57
6	2	34	62
7	0	36	62
8	0	35	58
9	2	35	57
10	2	39	61
11	7	35	62
12	0	38	57
13	2	34	63
14	0	36	51
15	2	37	52
Percentage	1.9%	36%	58%
SD	1.9	1.5	3.8

## Discussion

The most interesting finding of the present study is that new osteogenesis was observed in the grafted sites without showing remnants of the material. In all specimens no foreign body reaction or inflammatory infiltrate were present, thus confirming the high osteoconductivity of this biomaterial (Berardinelli et al., 2013). Indeed, the used biomaterial shows an almost complete resorption and a gradual apposition of newly formed bone (Grigolo et al., 2011; Berardinelli et al., 2013; Mozzati et al., 2017), while, 6 months after surgery, the use of other graft materials may show the presence of high quantity of residual graft particles (Scarano et al., 2012). In the early stages of healing, serving as a scaffold to guide effective bone regeneration, MgHA/collagen is resorbed by enzymes and cellular action over a period of 6–8 months (Scarano et al., 2017a). Usually, the bone graft is incorporated into the host site by means of interdigitation of the new bone deposited by the native bone (Scarano et al., 2006).

The ideal bone substitute should not evoke any adverse inflammatory response and be biocompatible, osteoconductive, osteoinductive and completely resorbable. Different osteoconductive biomaterials have been tested for maxillary sinus regeneration but, due to the absence of a complete resorption, local osteoprogenitor cells and poor wettability, months are required in order to have complete bone regeneration at the site of sinus floor elevation (Scarano, 2017). Clinical success has been obtained by grafting the maxillary sinus with different bone replacement materials before or simultaneously with implants placement. All surgical treatments in the maxillary region require detailed knowledge of anatomy and possible anatomical variations in order to avoid pitfalls, as well as an accurate preoperative diagnosis. Equally important is knowledge of the biological behavior of the biomaterials used to fill the maxillary sinus.

In fact, the nature of graft materials plays important roles in bone healing and regeneration. A biomaterial used in bone regeneration should have the following characteristics:

1. optimized structure for bone integration;
2. congruous pore volume to receive cells involved in tissue repairing;
3. enough pore interconnectivity with larger pore dimension for continuous tissue growth;
4. mechanical properties similar to the missing tissue;
5. facility in placement into the bone defect within a short setting time.

Bone substitute materials are available in different shapes and sizes, but they require major healing periods in comparison to autologous bone due to the reduced biological potential, as they are cell-free (Scarano et al., 2006).

Tissue engineering has allowed to successfully use osteoconductive biomaterials as carriers for growth factors and mesenchymal stem cells to increase tissue regeneration, accelerate osseointegration of dental implants and bone formation. Recently, the synergic effect of bone marrow stromal stem cells have been incorporated into a scaffold of porcine bone block, showing bone formation in surgical bone defects of the edentulous mandible in mini-pigs (Scarano et al., 2017b). However, these techniques have not been used yet in clinical practice, since bone substitute materials are cell-free and usually used in bone regeneration. On the other hand, the MgHA/collagen association represents a collagen-hydroxyapatite composite bone substitute, structured for bone regeneration on macro, micro and nano-scales, which increases the wettability. Indeed, this biomaterial graft quickly adsorbs the blood molecules and cells (Figure 2), thus promoting bone formation. The platelet growth factors and the combination of biomaterials were used with success for bone regeneration (Ohayon et al., 2016) and soft tissue augmentation (Scarano et al., 2016). The platelets imbibed in the MgHA/collagen lead to the activation and development of pseudopod aggregation and, ultimately, platelet degranulation. Alpha granules within the platelets release, via exostosis, a multitude of GFs which act as chemo attractants and mitogenic agents. The growth factors released by platelets within the biomaterial lead to appropriate wound healing. Absorption of the blood subsequent to bleeding, ending with the formation of fibrin or a blood clot, may stimulate cells with osteogenic, and probably angiogenic, potential to migrate to the surgical site. Also hydroxyapatite and collagen type I (HA/Collagen) have been used as a composite material and are found to enhance osteoblast differentiation (Geissler et al., 2000; Xie et al., 2004) and to accelerate osteogenesis (Serre et al., 1993).

Many biomaterials used for bone regeneration, a mixed bone/graft is obtained with a different metabolism for the turnover of native bone.

The outcome of the present study showed that an MgHA/collagen-based scaffold is completely resorbable. The combination of type I collagen (30%) and MgHA (70%) based scaffold mimicked the complex, hierarchically organized bone structure and improved bone formation.

Histologic evaluation of the newly formed tissues in sinus augmentation procedures is very helpful in understanding issues such as the nature and amount of newly formed bone and remnants of graft material. The tomograph showed the cortication of the buccal window in 13 sinuses, while in the 2 remaining sinuses, bone consolidation on the buccal aspect was evident, but no evidence of cortication was seen. Bone cortication in the wall window confirms the good healing of the entire bone graft. All sinuses healed without complications or clinical signs of sinusitis. The mean volume immediately after graft elevation and after 6 months remained stable and there were no significant differences. This outcome confirms that the bone regeneration obtained is stable up to 6 months, but a long-term follow-up is necessary. The outcome of the present study showed that the MgHA/collagen-based scaffold is a highly biocompatible biomaterial and completely resorbable (Babiker et al., 2012).

In the present study the percentage of marrow spaces was higher than that reported (Scarano et al., 2006) with other biomaterials, in which 40 ± 3.8% was found after 6–8 months. These differences could be related to the complete resorption of the biomaterial used in this study. Probably, the dimension of the particles of HA and Mg, and even the collagen, are beneficial for bone healing and graft resorption.

The shape and complete resorption of a biomaterial has many advantages: absence of foreign body reaction in case of perimplantis; facilitation of the repair of the Schneiderian membrane in case of drilling; production of only native bone and not bone/graft mix; easily adaptation to the dimension and shape of the sinus without risk of perforating the Schneiderian membrane. Our study also shows that grafted sites lead to woven bone and a large marrow space with the ability to adapt to the implant load. In this case, the dental implant placement will come into contact with only the native bone and not with the biomaterial, i.e., assuring an interface with physiological adaptation and bone remodeling, and improved peri-implant bone volume and interfacial loading strength. This is the first clinical study to document bone regeneration with MgHA/collagen-based scaffold in sinus lifting procedure.

However, the characteristics of design and methodology of this study have limitations that mean that the results cannot be considered conclusive. Nevertheless, these results help to set practice parameters that will assure a comparative study with a large number of patients in future research.

Short term histological and histomorphometrical evaluations with a larger number of patients will be necessary for a better comprehension of the resorption phase of this biomaterial. The main advantages of the combination of type I collagen (30%) and MgHA (70%) based scaffold is the complete resorption, also the mechanical properties allow it to be shaped and adapted, thus promoting bone regeneration in non-space maintaining defects.

In conclusion, this first clinical study found that MgHA/collagen-based scaffold can be successfully used for sinus augmentation procedures.

## Author contributions

AS Wrote paper and surgery. FL, GS, AT, BS performed data analysis. CM reviewed the manuscript.

### Conflict of interest statement

The authors declare that the research was conducted in the absence of any commercial or financial relationships that could be construed as a potential conflict of interest. The reviewer AP and handling Editor declared their shared affiliation, and the handling Editor states that the process met the standards of a fair and objective review.
